# Drug resistance gene expression and chemotherapy sensitivity detection in Chinese women with different molecular subtypes of breast cancer

**DOI:** 10.20892/j.issn.2095-3941.2020.0157

**Published:** 2020-12-15

**Authors:** Jing Zhao, Hailian Zhang, Ting Lei, Juntian Liu, Shichao Zhang, Nan Wu, Bo Sun, Meng Wang

**Affiliations:** 1 Department of Breast Cancer, Tianjin Medical University Cancer Institute and Hospital, National Clinical Research Center for Cancer, Key Laboratory of Cancer Prevention and Therapy, Tianjin, Tianjin’s Clinical Research Center for Cancer, Tianjin 300060, China; 2 Department of Pathology, West China Hospital, Sichuan University, Chengdu 610041, China

**Keywords:** Breast cancer, molecular subtype, CD133, drug resistant gene, chemosensitivity

## Abstract

**Objective:**

The aim of the study was to identify specific chemosensitivity drugs for various molecular subtypes of breast tumors in Chinese women, by detecting the expression of drug resistance genes and by using the drug sensitivity test on different molecular subtypes of breast cancers.

**Methods:**

The expression of drug resistance genes including *Topo II, GST-*π*, P-gp, LRP,* and* CD133* were detected with immunohistochemistry in a tissue microarray. Drug sensitivity tests included those for paclitaxel, epirubicin, carboplatin, vinorelbine, and fluorouracil and were conducted on primary cancer tissue cells and cell lines, including the T47D, BT-474, and MDA-MB-231 cells and human breast cancer xenografts in nude mice.

**Results:**

The different drug resistant genes *Topo II, GST-*π*, P-gp,* and* LRP* were differentially expressed among different molecular subtypes of breast cancers (*P* < 0.05). Positive expression of CD133 was highest in basal-like breast cancer (*P* < 0.05). Kaplan-Meier survival analysis showed that positive expressions of Topo II and CD133 both correlated with shorter disease-free survival (DFS) (*P* < 0.05) and overall survival (*P* < 0.05), and positive expression of LRP correlated only with shorter DFS (*P* < 0.05). BT-474 showed chemosensitivity to paclitaxel and epirubicin, while MDA-MB-231 showed chemosensitivities to paclitaxel, epirubicin, carboplatin, and fluorouracil (T/C ≤ 50%). The basal-like and HER2+ breast cancer primary cells showed chemosensitivities to paclitaxel and epirubicin with significant differences compared with luminal breast cancer primary cells (*P* < 0.05).

**Conclusions:**

The differential expression of drug resistance genes and the differential chemosensitivities of drugs in different molecular subtype of breast cancers suggested that individual treatment should be given for each type of breast cancer.

## Introduction

Breast cancer is a highly heterogeneous malignant tumor^1^. In 2000, Perou et al.^2^ first proposed the concept of molecular classification based on gene expression profiling, which divided breast cancer into luminal (including luminal A and luminal B), HER2-positive (HER2+), basal-like, and normal-like subtypes. Different molecular subtypes of breast cancers showed their own unique biological characteristics, epidemiological characteristics, and sensitivities to drug treatments^3,4^. In clinical practice, individualized treatment has been used in endocrine therapy, and targeted therapy for breast cancer according to the molecular subtypes of breast cancer^5,6^. Due to the lack of an effective judgment index for chemotherapy, individualized chemotherapy regimens for each patient have not been established. Selection of the chemotherapy regimen is still according to traditional pathological parameters for different molecular types of breast cancers. This treatment regimen not only causes a waste of healthcare resources, but also results in unnecessary side effects to patients, and even leads to multi-drug resistance of the tumor, which presents difficulties for further patient treatments^7,8^. Besides the above reasons, stem cell drug resistance may be another reason for chemotherapy failure^9^. It is therefore important to find personalized and effective chemotherapy regimens.

A large-scale sample analyses for the expressions of drug resistance genes in Chinese women with different molecular subtypes of breast cancer has not been done. In the present study, the expressions of drug resistance genes and CD133 in different molecular subtypes of breast cancer were therefore detected with immunohistochemistry (IHC) in a tissue microarray (TMA) from a large cohort. Furthermore, cell lines and primary cells with different molecular subtypes were subjected to drug sensitivity tests to identify the factors responsible for mediating chemosensitivity. The results of the present study provide a reference for the choice of specific chemotherapy regimens for each subtype of Chinese breast cancer patient. Although this study has been conducted in Chinese women, is it possible that the results could be relevant to women from other backgrounds.

## Materials and methods

### Patients and follow-up

We obtained 600 mastectomy specimens from the Tianjin Medical University Cancer Institute and Hospital from 2013 to 2014. All specimens were diagnosed with invasive ductal carcinoma with no specific type. None of the patients received preoperative treatment. Clinic pathological variables such as age, sex, histological grading, lymph node metastasis, status of extranodal extension and distant metastasis, pathological stage, and follow-up time were evaluated. The results of immunohistochemical markers including estrogen receptor (ER), progestogen receptor (PR), epidermal growth factor receptor 2 (HER2), cytokeratin5/6 (CK5/6), epidermal growth factor receptor (EGFR), and Ki-67 were obtained from the pathological reports.

### Tissue microarrays

Hematoxylin and eosin staining of the samples was initially reviewed, and the representative areas were marked in the paraffin blocks, avoiding necrotic and hemorrhagic areas. TMA blocks selected from these marked regions using a specialized manual arraying instrument (Model MTA1, Beecher Instruments, Sun Prairie, WI, USA) were constructed in duplicates for each case at a core diameter of 2 mm and a density of 60 cores per block. In total, 10 array blocks were prepared during the study. In addition, 20 normal breast tissue blocks from 2014 were randomly chosen.

### Immunohistochemistry (IHC)

After dewaxing and hydration, 4 μm sections from formalin-fixed and paraffin-embedded tissues were treated with an ethylenediaminetetraacetic acid (EDTA) buffer solution (pH 9.0) using pressure cooking for 2 minutes. Endogenous peroxidase activity was blocked following incubation in 3% H_2_O_2_ for 10 minutes. Any nonspecific binding sites were blocked with 10% normal goat serum for 5 minutes. Tissue sections were incubated with primary antibodies against P-gp (1:40; Zymed, South San Francisco, CA, USA), LRP (1:75; Zymed), Top II (1:100; Zymed), and CD133 (1:100; Zymed). There was no need to perform antigen retrieval for GST-π (1:100; Zymed). All sections were sequentially treated with a biotinylated anti-rabbit or anti-mouse immunoglobulin for 20 minutes at 37 °C and peroxidase-labeled streptavidin for 20 minutes at 37 °C. A negative control with phosphate-buffered saline (PBS) was included. All sections were counterstained with 3,3′-diaminobenzidine tetrahydrochloride. The scoring of expression profiles was performed by at least 2 independent investigators. Cases were considered positive for P-gp, LRP, and GST-π expressions upon observation of cytoplasmic immunoreactivity in tumor cells. The immunoreactivity scores of P-gp, LRP, and GST-π were evaluated according to the signal intensity (0, no reactivity; 1, weak reactivity; 2, strong reactivity) and percentage of positive cells (0, 0% reactive cells; 1, < 25% reactive cells; 2, 25%–49% reactive cells; 3, 50%–74% reactive cells; 4, ≥ 75% reactive cells). Each case was graded by the addition of the scores as 0(-), 1–2(+), 3–5(++), 6–8(+++). A value of 0 represented negative and 1–8 represented positive. The expression of Topo II was observed in cell nuclei, and CD133 was observed either in the cytoplasm or cell membrane, and the standard of the immunoreactivity score was the same as previously described^10^.

### Cell lines and cell culture

The human mammary epithelial cell lines, T47D, BT-474, and MDA-MB-231, were obtained from the Cancer Hospital of Tianjin Medical University. The cell lines were cultured in Roswell Park Memorial Institute-1640 medium (Gibco, Gaithersburg, MD, USA) supplemented with 10% fetal bovine serum (Gibco) at 37 °C in a humidified atmosphere with 5% CO_2_.

### Western blot

The T47D, BT-474, and MDA-MB-231 cell lines were harvested, washed once with PBS, and resuspended in lysis buffer (50 mM Tris-HCl, 1 mM EDTA, 150 mM NaCl, 0.1% SDS, 0.5% deoxycholic acid, 1% NP-40, 2.0 μg/mL aprotinin, 0.02% sodium azide, and 1 mM phenylmethylsulphonyl fluoride) on ice. Then, a lysis buffer was added and the supernatant was collected. Total proteins (80 μg) were subjected to SDS-PAGE. ER, PR, and Cerb-B2 protein antibody (ab40839; Abcam, Cambridge, UK) combined with goat anti-rabbit IgG conjugated with horseradish peroxidase was chosen, and β-actin antibody (AF1186; Beyotime Biotechnology, Beijing, China) was used as a loading control.

### Extraction and cultivation of primary breast cancer cells

The tumor tissues were cut into small pieces (< 1 mm) with a blade, and mixed with Hank’s solution. The tissues were washed 2–3 times with Hank’s solution and centrifuged at 1,000 × *g* for 3 minutes. The supernatants were discarded and a few drops of blood were added to the tissues to allow absorption of blood into the tissues. The samples were placed in a small flask and the bottom of the bottle was carefully monitored. Approximately 4–5 mL of medium was gently added to expose the cells from the edges of the bottle, and the incubation was continued at 37 °C in an incubator for 2–3 hours.

### Animal experiments

Experimental animal studies were approved by the Tianjin Medical Animal Care and Use Committee. Female BALB/c athymic nude mice were used in this study. BALB/c athymic nude mice were injected with 5 × 10^6^ T47D, BT-474, or MDA-MB-231 cells into the left dorsal flank^11^. BALB/c athymic nude mice were monitored for tumor development by weekly mammary gland palpation, and tumor volumes were determined using a caliper. Mice were sacrificed when primary tumors reached a maximum volume of 2.5 cc, then the tumor tissue was harvested.

### The collagen gel droplet-embedded culture drug sensitivity test (CD-DST) to evaluate sensitivity to chemotherapy drugs

Preparation of the tumor cell suspension. The excisional specimen including primary breast cancer tissues and tumor tissues from nude mice were finely minced using scissors, suspended in Hank’s balanced saline solution (HBSS; Gibco), treated with Dispersion Enzyme Cocktail EZ (including 1.0% collagenase; Nitta Gelatin, Osaka, Japan) and digested at 37 °C for 1 hour. The dispersed tumor cells were collected by centrifugation at 1,000 rpm for 3 minutes, filtered through a 308 nm nylon mesh, washed in HBSS, suspended in PCM-1 medium (Nitta Gelatin), and then incubated in a collagen gel-coated flask (CG-flask; Nitta Gelatin) in a CO_2_ incubator at 37 °C for 24 hours. The collagen gel in the CG-flask was dissolved in a cell dispersion using EZ, and only viable cells that adhered to the collagen gel were collected and used for the sensitivity test.

The collagen gel droplet embedded culture-drug sensitivity test. Type I collagen, 10× F-12 medium, and reconstitution buffer (Cellmatrix Type CD; Nitta Gelatin) were combined in an ice bath at a ratio of 8:1:1. The prepared tumor cell suspension was added to a collagen solution (1:10, v:v) at a final density of 2 × 10^5^ cells/mL. A total of 3 drops of the collagen-cell mixture (30 μL/drop) were placed in each well of a 6-well multiplate on ice and allowed to gel at 37 °C in a CO_2_ incubator; the final concentration was ~3 × 10^3^ cells/droplet. One hour later, each well was overlaid with 3 mL Dulbecco’s Modified Eagle’s Medium/F 12 medium (Gibco) containing 10% fetal bovine serum (Gibco, Grand Island, NewYork, USA), and incubated in a CO_2_ incubator at 37 °C overnight. Paclitaxel, epirubicin, carboplatin, vinorelbine, were then added to final concentrations of 0.2 and 2.0 μg/mL, 0.02 and 0.2 μg/mL, 0.01 and 0.1 μg/mL, and 1.0 and 10 μg/mL, respectively, followed by further incubation for 24 hours. IFO (1 or 10 μg/mL) was added as a negative control.

*In vitro* chemosensitivity test. The primary breast cancer cells and the T47D, BT-474, and MDA-MB-231 cell lines removed from the mice were embedded in an artificial extracellular matrix type I collagen gel and cultured in a three-dimensional model, which was similar to the microenvironment *in vivo*. The appropriate concentrations (calculated from the drug plasma concentration time curve) of chemotherapeutic agents (paclitaxel: 0.1 μg/mL; epirubicin: 1.0 μg/mL; carboplatin: 1.0 μg/mL; vinorelbine: 0.05 μg/mL; and fluorouracil: 1.0 μg/mL) were used to treat primary breast cancer cells, and the cell lines were extracted from the mice after 24 hours. After removal of the medium containing the anticancer drugs, each well was rinsed twice with 3 mL HBSS, overlaid with 4 mL PCM-2 medium (Serum Free Medium; Nitta Gelatin), and incubated for an additional 7 days. On the fourth day of incubation, the medium was changed. At the end of the incubation, neutral red (Nitta Gelatin) was added to each well at a final concentration of 50 μg/mL, and cells in the collagen gel droplets were stained for 2 hours. Each collagen droplet was fixed with 10% neutral formalin buffer, washed in double-distilled water, air-dried, and quantified using image analysis. The *in vitro* sensitivity was expressed as the percentage of the T/C ratio, where T is the total volume in the treated group and C is the total volume in the control group. When the T/C ratio was ≤ 50%, the *in vitro* drug sensitivity was regarded as effective. T/C ratios of > 50% and ≤ 60% were considered borderline, and a T/C ratio of > 60% was considered to indicate a lack of drug efficacy.

### Statistical analysis

All the statistical analyses were performed using SPSS statistical software for Windows, version 19.0 (SPSS, Chicago, IL, USA). The expression of drug resistance genes in different molecular subtypes was analyzed using the chi-square test. Comparisons of the relationships between the expressions of different drug resistance genes and the clinical features of breast cancer were performed using the chi-square test. The Kaplan-Meier survival curve was used for survival analysis. The log-rank test was performed to compare the survival differences between subgroups. A value of *P* < 0.05 was considered statistically significant.

## Results

The 548 samples included 148 luminal A (ER+/PR+, HER2−, Ki-67<20%), 91 luminal B (ER+/PR+, HER2+/Ki-67≥20%), 113 HER2+ (ER-, PR-, HER2+), and 196 basal cell-like (ER−, PR−, HER2−, CK5/6+ and/or EGFR+) breast cancers.

### Relationship between expressions of different drug resistance genes and pathological variables

**Figure 1:**
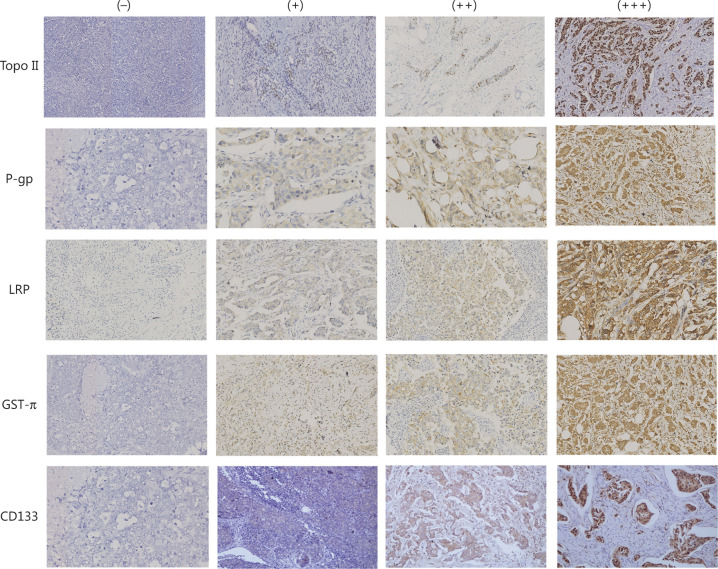
Immunohistochemistry for drug-resistant genes and CD133 (200×). The immunohistochemistry (IHC) results of Topo II: (−), (+), (++), (+++); the IHC results of P-gp: (−), (+), (++), (+++); the IHC results of LRP : (−), (+), (++), (+++); the IHC results of GST-π:(−), (+), (++), (+++); and the IHC results of CD133: (−), (+), (++), (+++).

The correlation of the expressions of different drug resistance genes and clinical pathological factors including age of onset, tumor size, histological grade, lymph node metastasis, lymphatic thrombus, and clinical stage of patients with primary invasive breast cancers were analyzed. Topo II positive expression significantly correlated with histological grades (*P* = 0.01), but there was no correlation with the age of onset, tumor size, lymph node metastasis, lymphatic thrombus, or clinical stage (*P* > 0.05). The positive expression of LRP, GST, and P-gp had no correlation with the histological grade, tumor size, or clinicopathological parameters (*P* > 0.05) (**Table 1**) (**Figure 1**).

**Table 1:** Relationship between different drug-resistant genes and clinicopathological variables of breast cancers

	Total	Topo II		*P*	LRP		*P*	GST-π		*P*	P-gp		*P*
		− (%)	+ (%)		− (%)	+ (%)		− (%)	+ (%)		− (%)	+ (%)	
Histological grade				0.001*			0.78			0.45			0.08
I	16	14 (87.5)	2 (12.5)		8 (50.0)	8 (50.0)		6 (37.5)	10 (62.5)		8 (50.0)	8 (50.0)	
II	336	150 (44.6)	186 (55.4)		176 (52.3)	160 (47.6)		162 (48.2)	174 (51.8)		149 (44.3)	187 (55.7)	
III	196	81 (41.3)	115 (58.7)		90 (45.9)	106 (54.1)		86 (43.9)	110 (56.1)		68 (34.7)	128 (65.3)	
Vessel thrombus				0.89			0.17			0.47			0.43
Negative	500	211 (42.2)	289 (57.8)		246 (49.2)	244 (48.8)		242 (48.4)	258 (51.6)		202 (40.4%)	298 (59.6%)	
Positive	48	34 (70.8)	14 (29.2)		28 (58.3)	20 (42.7)		22 (45.8)	26 (54.2)		23 (47.9%)	25 (52.1%)	
Clinical stage				0.73			0.14			0.62			0.90
I	37	15 (40.5)	22 (59.5)		14 (37.8)	23 (62.2)		11 (29.7)	26 (60.3)		16 (43.2)	21 (56.8)	
II	359	157 (43.7)	202 (56.3)		180 (50.1)	179 (49.9)		162 (45.1)	197 (54.9)		143 (39.8)	216 (60.2)	
III	152	73 (48.0)	79 (52.0)		80 (52.6)	72 (47.4)		81 (53.3)	71 (46.7)		66 (43.4)	86 (56.6)	
Lymph node metastasis				0.73			0.99			0.55			0.80
Negative	230	104 (45.2)	126 (54.8)		114 (49.6)	116 (50.4)		101 (43.9)	129 (56.1)		91 (39.6)	139 (60.4)	
Positive	318	141 (44.3)	177 (55.7)		160 (50.3)	158 (49.7)		153 (48.1)	165 (51.9)		134 (42.1)	184 (57.9)	
Age (mean ± SD)				0.5			0.15			0.66			0.33
		52.9±6.7	52.1±5.8		51.2±7.9	53.9±5.8		52.7±6.7	52.2±5.9		51.8±8.6	52.9±6.2	
Tumor size (mean ± SD)				0.49			0.28			0.17			0.84
		3.0±2.5	3.0±2.7		3.1±2.7	2.9±2.3		3.1±3.0	2.9±2.6		3.0±2.7	2.9±2.8	

a The Fisher test was used when *N* < 5; *P < 0.05.

### Expression of drug resistance genes in different molecular subtypes

The overall positive expression percentage of Topo II was 55.3% (303/548) in our study with 43.9% (65/148) in luminal A, 52.7% (48/91) in luminal B, 60.2% (68/113) in HER2+, and 62.2% (122/196) in the basal-like subtype, which were statistically significant (χ^*2*^= 12.906, *P* = 0.005) (**Table 2**).

**Table 2:** Expression of different drug resistance genes and CD133 in different molecular subtypes of breast cancers

Drug-resistance gene	Total	Molecular subtype				χ^2^	*P*
		Luminal A (%)	Luminal B (%)	HER2+(%)	Basal-like (%)		
Topo II						12.906	0.005*
+	303	65 (21.5)	48 (15.8)	68 (22.4)	122 (40.3)		
–	245	83 (33.9)	43 (17.5)	45 (18.4)	74 (30.2)		
P-gp						19.9947	<0.001*
+	323	89 (27.6)	55 (17.0)	47 (14.6)	132 (40.9)		
–	225	59 (26.2)	36 (16.0)	66 (29.3)	64 (28.4)		
LRP						18.601	<0.001*
+	274	86 (31.4)	46 (16.8)	67 (24.5)	75 (27.4)		
–	274	62 (22.6)	45 (16.4)	46 (16.8)	121 (44.2)		
GST-π						14.183	0.003*
+	294	85 (28.9)	46 (15.6)	49 (16.7)	74 (25.2)		
–	254	63 (24.8)	45 (17.7)	64 (25.2)	122 (48.0)		
CD133						46.839	<0.001*
+	278	50 (18.0)	36 (13.0)	57 (20.5)	135 (48.6)		
–	270	98 (36.3)	55 (20.4)	56 (20.7)	61 (22.6)		

*P < 0.05.

The overall positive expression percentage of P-gp was 58.9% (323/548) in our study, with 60.1% (89/148), 60.4% (55/91), 41.6% (47/113), and 67.35% (132/196) in luminal A, luminal B, HER2+, and the basal-like subtypes, respectively, which were statistically significant (χ^*2*^= 19.947, *P* < 0.001) (**Table 2**).

For LRP, the overall positive expression percentage was 50% (274/548) in our study. with 58.1% (86/148) in luminal A, 50.5% (46/91) in luminal B, 59.3% (67/113) in HER2+, and 38.3% (75/196) in the basal-like subtypes, which were statistically significant (χ^*2*^ = 18.601, *P* < 0.001) (**Table 2**).

The overall positive expression rate of GST-π was 53.6% (294/548) in our study, and 57.4% (85/184), 50.5% (46/91), 43.4% (49/113), and 37.8% (74/196) positive expression percentages were observed in luminal A, luminal B, HER2+, and basal-like subtype, respectively, which were statistically significant (χ^*2*^ = 14.183,* P* = 0.003) (**Table 2**).

### Relationship between the expression of different drug resistance genes and the prognoses

**Figure 2:**
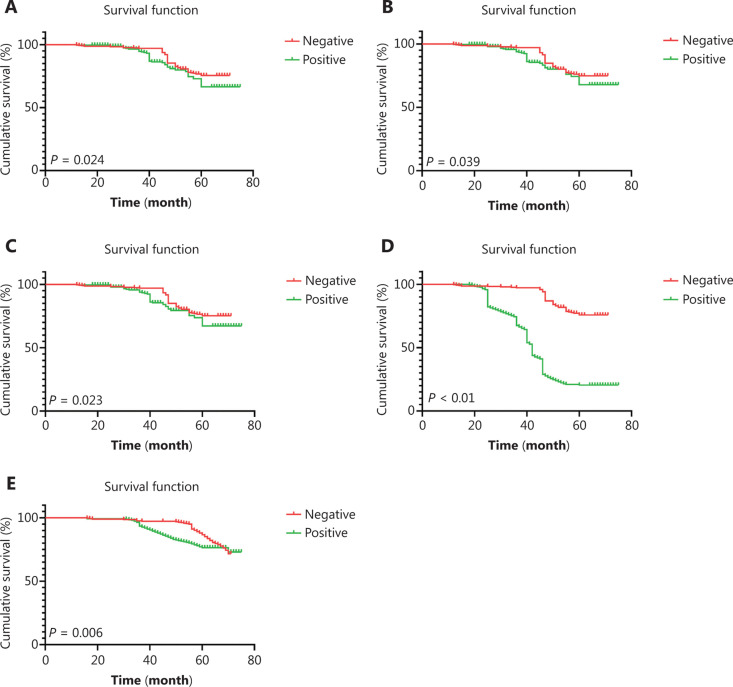
The survival curve of drug resistance gene expression in breast cancer patients. (A) The disease-free survival (DFS) curve of Topo II, *P* = 0.024; (B) the overall survival (OS) survival curve of Topo II, *P* = 0.039; (C) the DFS survival curve of p-gp, *P* = 0.023; (D) the DFS survival curve of CD133, *P* < 0.001; and (E) the OS survival curve of CD133, *P* = 0.006.

The median follow-up times for disease-free survival (DFS) and overall survival (OS) were 46 months (13–74 months) and 52 months (17–74 months), respectively. In the positive groups for Topo II, P-gp, LRP, and GST-π expressions, the OS percentages were 81.9%, 86%, 82.3%, and 84.7%, respectively, while the DFS percentages were 76.5%, 80.2%, 76.9%, and 79.8%, respectively. The positive expression of Topo II significantly correlated with shorter OS and DFS (*P* = 0.024 and *P* = 0.039, respectively) (**Figure 2A and 2B**), while the positive expression of P-gp had a shorter DFS (*P* = 0.023) (**Figure 2C**).

### Expression and survival analysis of CD133 in different molecular subtypes of breast cancers

The overall positive expression percentage of CD133 was 50.7% (278/548), with 33.7% (50/148) in luminal A, 39.5% (36/91) in luminal B, 50.7% (57/113) in HER2+, and 68.9% (135/196) in the basal-like subtype, which were statistically significant (χ^*2*^ = 46.839, *P* < 0.001). Survival analysis revealed that the positive expression of CD133 significantly correlated with shorter DFS and OS (*P* < 0.001 and *P* = 0.006, respectively) (**Figure 2D and 2E**).

### Determination of the molecular subtypes of different breast cancer cell lines

**Figure 3:**
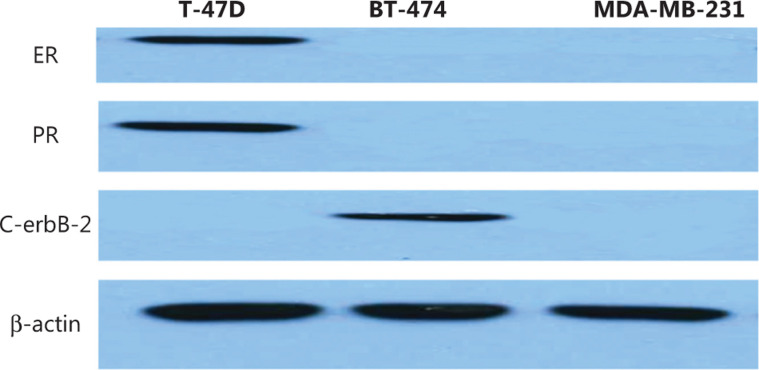
The confirmation of different molecular subtypes of breast cancer cell lines using Western blot.

The immunophenotypes of MDA-MB-231 was ER−, PR−, HER2−, BT-474 was ER−, PR-, HER2+, and T47D was ER+, PR+, HER2−. In this experiment, the cell proteins were extracted and the cellular molecular phenotypes were confirmed using Western blot (**Figure 3**).

### Detection of the drug sensitivities of different molecular subtypes of breast cancers with CD-DST

In our studies, 224 cases of fresh breast cancer tissues were successfully cultured, and their chemosensitivities were evaluated based on their molecular subtypes. The chemosensitivities to paclitaxel in luminal A, luminal B, HER2+, and the basal-like were 34.4% (40/116), 36.4% (8/22), 48.0% (12/25), and 56.9% (33/58), respectively (χ^*2*^ = 8.626, *P* = 0.035), and the chemosensitivities to epirubicin were 31.4% (37/118), 34.8% (8/23), 75% (18/24), and 45.6% (27/59) in luminal A, luminal B, HER2+, and the basal-like subtype, respectively (χ^*2*^ = 16.973, *P* = 0.001) (**Table 3**).

**Table 3:** The collagen gel droplet-embedded culture drug sensitivity test of different chemotherapy drugs in primary cells

Chemotherapy drug	Total	Molecular subtype				χ^*2*^	*P*
		Luminal A (%)	Luminal B (%)	HER2+ (%)	Basal-like (%)		
Paclitaxel						8.626	0.035*
+	93	40 (54.8)	8 (11.0)	12 (16.4)	33 (45.2)		
–	128	76 (59.4)	14 (10.9)	13 (10.2)	25 (19.5)		
Epirubicin						16.973	0.001*
+	90	37 (41.1)	8 (8.9)	18 (20.0)	27 (30.0)		
–	134	81 (60.4)	15 (11.2)	6 (4.5)	32 (28.9)		
Carboplatin						7.732	
+	62	30 (48.4)	2 (3.2)	9 (14.5)	21 (33.9)		
–	142	79 (55.6)	18 (12.7)	15 (10.6)	30 (21.1)		0.052
Vinorelbine						1.958	
+	46	28 (60.9)	4 (8.7)	5 (10.9)	9 (19.6)		
–	164	82 (50.0)	17 (10.4)	19 (11.6)	46 (28.0)		0.581
Fluorouracil						4.384	
+	33	20 (60.6)	1 (3.0)	3 (9.1)	9 (27.3)		
–	104	50 (48.1)	15 (14.4)	10 (9.6)	29 (27.9)		0.223

*P < 0.05.

### Detection of the drug sensitivities of different molecular subtypes of breast cancer cell lines with CD-DST

**Figure 4:**
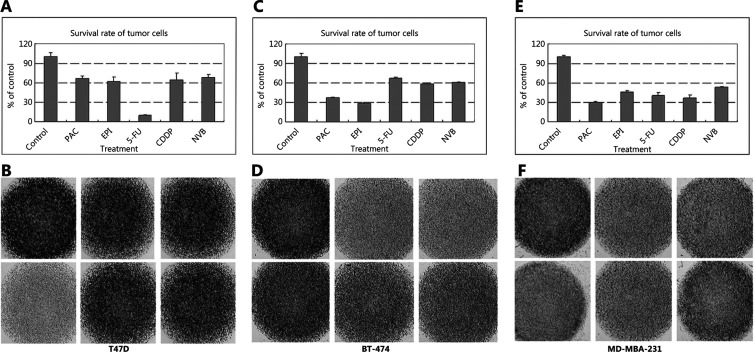
The sensitivities of (A, B) T47D, (C, D) MDA-MB-435, and (E, F) MDA-MB-231 cells to paclitaxel, epirubicin, carboplatin, vinorelbine, and fluorouracil. The T47D cell line was only sensitive to fluorouracil, while MDA-MB-435 cells showed chemosensitivities to paclitaxel and epirubicin. MDA-MB-231 cells were sensitive to paclitaxel, epirubicin, carboplatin, vinorelbine, and fluorouracil. Images B, D, and F: The *in vitro* sensitivity was expressed as the percentage of the T/C ratio, where T is the total volume in the treated group and C is the total volume in the control group. When the T/C ratio was ≤ 50, the *in vitro* drug sensitivity was regarded as effective. A T/C ratio of > 50 and ≤ 60 was considered borderline, and a T/C ratio of > 60 was considered to indicate a lack of efficacy. The image in the top left corner represents the control. From the left to right in the first line were controls, paclitaxel, epirubicin, and in the second line were carboplatin, vinorelbine, and fluorouracil which was shown in the B, D and F. And the corresponding results were shown in the A, C and E. The darker the color, the more cells were alive. Images A, C, and E represent the survival of T47D, BT-474, and MDA-MB-231 cells exposed to different chemotherapy drugs.

We detected the sensitivities of T47D, BT-474, and MDA-MB-231 cells to paclitaxel, epirubicin, carboplatin, vinorelbine, which were extracted from the T47D, BT-474, and MDA-MB-231 human breast cancer xenografts in nude mice (**Supplementary Figure S1**). The T47D cell line was only sensitive to fluorouracil (**Figure 4A and 4B**), while BT-474 cells showed chemosensitivities to paclitaxel and epirubicin (**Figure 4C and 4D**). MDA-MB-231 cells were sensitive to paclitaxel, epirubicin, carboplatin, vinorelbine, and fluorouracil (T/C ≤ 50%) (**Figure 4E and 4F**) (**Table 4**).

**Table 4:** The collagen gel droplet-embedded culture drug sensitivity test of different chemotherapy drugs in cell lines

Chemotherapy drug	T47D		BT-474		MDA-MB-231	
	Survival rate (%)	Standard deviation	Survival rate (%)	Standard deviation	Survival rate (%)	Standard deviation
Paclitaxel	67.08	6.49	37.08	1.22	29.53	1.61
Epirubicin	62.48	6.24	28.28	1.02	45.23	2.25
Carboplatin	64.9	10.20	58.76	0.92	36.15	4.82
Vinorelbine	68.48	4.45	60.76	1.00	53.02	1.00
Fluorouracil	10.05	0.54	67.09	1.23	40.60	4.10

Chemosensitivity (T/C ≤ 50%); chemoresistant (T/C > 50%).

## Discussion

In clinical practice for breast cancer, the application of endocrine therapy and targeted therapy is selected according to the molecular typing of the breast cancer^12^. Chemotherapy, as the most important treatment for breast cancer, currently lacks effective molecular indicators. Selection of the chemotherapy regimen is still according to the traditional pathological parameters for different molecular breast cancers. Taxanes and anthracyclines are the first-line chemotherapy treatment for breast cancer, while platinum and vinblastine are often used as the second-line drugs in clinical practice^13,14^. Carey et al^15^ compared the efficacies of doxorubicin and cyphamide in neoadjuvant chemotherapy for different subtypes of breast cancers. The results showed that there were significant differences in the clinical response percentages and pathological responses of different subtypes of breast cancers, in which HER2+ and basal-like breast cancer showed strong chemotherapy sensitivities. Therefore, individualized chemotherapy based on molecular typing has an important role in the treatment of breast cancers.

Several factors are known to affect drug resistance, including the efflux of intracellular drugs, metabolic detoxification, changes in drug target molecules, DNA repair ability, and apoptosis regulation^16^. In addition to these changes, some studies^17,18^ have found that the development of drug resistance is accompanied by changes in the expressions of drug resistance genes that are deemed as the most important factor. Based on the molecular subtypes of breast cancer, the expression of common drug resistance genes was detected and compared in our study.

High positive expression rate and strong expression intensity of Topo II protein in basal-like and HER2+ breast cancer suggested that anthracycline drugs were sensitive to basal-like and HER2+ breast cancers, which was consistent with previous reports^19,20^. The high expression of P-gp and low expression of LRP and GST-π in basal-like breast cancer indicated that basal-like breast cancer may be sensitive to platinum drugs, while it may be resistant to vinca alkaloids drugs. The low expression of Topo II protein expression and high expressions of LRP and GST-π protein in luminal breast cancer indicated the poor chemotherapy sensitivity of this type of breast cancer.

IHC results provided preliminary evidence for the sensitivity of different molecular subtypes of breast cancer to chemotherapeutics. We verified the reliability of these results in breast cancer cell lines and primary cells. It has been reported^21,22^ that there is some difference in the gene copy number variation between cell lines and primary tumors in different subtypes of breast cancers, while the response of cell lines to chemotherapy is similar to that of primary tumors.

We detected the sensitivity of different cell lines to chemotherapeutics drugs, and found that T47D cells, which represented luminal breast cancer, were resistant to the majority of chemotherapy drugs, while BT474, representative of the HER2+ breast cancer and MDA-MB-231 representative of basal-like breast cancer, were sensitive to paclitaxel. In the CD-DST for primary breast cancer cells, luminal breast cancer showed low sensitivity to paclitaxel, whereas the sensitivities of HER2+ and the basal-like subtypes showed high sensitivity to paclitaxel. HER2+ breast cancer showed the highest sensitivity to epirubicin in CD-DST primary breast cancer cells, while luminal A showed the lowest sensitivity to epirubicin. The immunohistochemical study evaluating LRP and GST-π expression in different subtypes of breast cancer suggested that basal-like breast cancer was sensitive to platinum. The drug sensitivity test with MDA-MB-231 cells confirmed this observation. Although the sensitivities of different subtypes of breast cancer to platinum were not statistically significant, the basal-like subtype was the most sensitive to platinum among all subtypes of breast cancer, reaching a value of 41.76%. This result also confirmed the reliability of the test results as a guide in clinical settings.

Combining the expression of drug resistance genes in different molecular subtype breast cancers with the chemotherapy sensitivities *in vivo*, we concluded that basal-like breast cancer showed sensitivity to taxanes, anthracycline, and carboplatin. Therefore, the treatment for basal-like breast cancer should include the above drugs for the baseline chemotherapy regimen. HER2+ breast cancer was the most sensitive to anthracycline drugs, followed by taxanes, so the chemotherapy regimen including the two types of drugs may be a preferred alternative. Luminal breast cancer did not show sensitivity to a variety of chemotherapy drugs, so the effect of chemotherapy for such patients is limited, and endocrine therapy is still the first-line treatment^23,24^.

The expression of different drug-resistant genes and proliferation-related genes determines the chemotherapy sensitivity of different subtypes of breast cancers. Although basal-like and HER2+ breast cancers showed higher chemosensitivities, the prognoses were poor, which may be related to the existence of tumor stem cells^25,26^. CD133, which is an overexpressed cancer stem marker, was associated with resistance to chemotherapy and poor prognoses^27^. In our study, a high expression of CD133 was detected in basal-like and HER2+ breast cancers, which was associated with poor prognoses. The existence of cancer stem cells results in a diversity of cells in different cell stages, which leads to tumor heterogeneity indicative of the varying phenotypes and proliferation potentials of tumors^28^. In the same tumors, cancer stem cells are more drug-resistant than non-cancer stem cells following conventional chemotherapy^29^. Targeted therapy for tumor stem cells besides the traditional chemotherapy regimens may achieve a better treatment effect.

Sufficient chemotherapy drugs and the full course of chemotherapy not only kills a large number of tumor cells, but also enriches stem cells with resistance to chemotherapy, which is the main cause for drug resistance and poor prognoses for breast cancer^26,30^. In the treatment for basal-like and HER2+ breast cancers, except for chemotherapy, targeted stem cell therapy should therefore be considered^31^.

In conclusion, we detected drug resistance genes in different molecular subtypes of breast cancer, which may provide a clue to chemosensitivity before clinical treatment. According to the different chemical sensitivities of different molecular subtypes, to obtain better therapeutic effects, in addition to the traditional chemotherapy regimen, targeted therapy for cancer stem cells should be added.
