# Supplementary material for: Drug resistance gene expression and chemotherapy sensitivity detection in Chinese women with different molecular subtypes of breast cancer
Source: Cancer Biol Med. 2020 Dec 15;17(4):1014–25. doi: 10.20892/j.issn.2095-3941.2020.0157 (PMC7721095; doi:10.20892/j.issn.2095-3941.2020.0157)
Supplement: Supplementary file 1 [file cbm-17-1014-s001.pdf]

Supplementary material

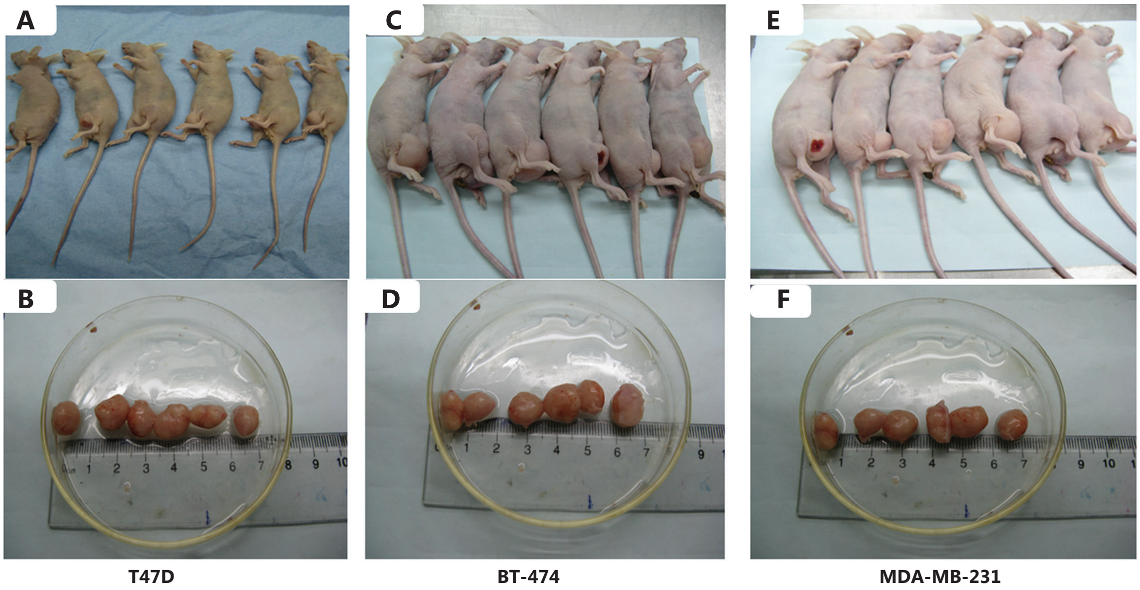

Figure S1 BALB/c athymic nude mice were injected with 5×10^6 (A, B) T47D, (C, D) BT-474, and (E, F) MDA-MB-231 cells into the left dorsal flank per mouse. BALB/c athymic nude mice were monitored for tumor development by weekly mammary gland palpation, and tumor volumes were determined using a caliper until the tumors reached a maximum volume of 2.5 cc (A, C, D). The tumor size according to the corresponding cell line was measured (B, E, F).
